# The impact of small-for-gestational-age Status on the outcomes in very-Low-birth-weight (VLBW) premature infants: a prospective cohort study in Taiwan

**DOI:** 10.3389/fped.2023.1209765

**Published:** 2023-07-14

**Authors:** Chia-Ying Lin, Hung-Yang Chang, Jui-Hsing Chang, Chyong-Hsin Hsu, Wai-Tim Jim, Chun-Chih Peng, Chia-Huei Chen

**Affiliations:** ^1^ Department of Pediatrics, MacKay Children’s Hospital, Taipei, Taiwan; ^2^ Department of Medicine, MacKay Medical College, New Taipei City, Taiwan; ^3^ Premature Baby Foundation of Taiwan, Taipei, Taiwan

**Keywords:** children development, preterm infants, SGA, small-for-gestational age, VLBW, very-low-birth-weight infant

## Abstract

**Background:**

The impact of small-for-gestational-age (SGA) on very-low-birth-weight (VLBW) premature infants remains inconclusive. This study aimed to assess the effects of being born SGA status on the short-term and long-term outcomes in VLBW preterm infants.

**Methods:**

We conducted a population-based, prospective cohort study on VLBW preterm infants born in Taiwan between 2012 and 2017. Sociodemographic, neonatal, growth and neurological data at 2 years of corrected age were collected. A total of 4243 VLBW infants born at 24 through 32 completed weeks' gestation participated in this study, of whom 1,005 had SGA status defined as a birth weight <10th percentile of gestation, and 3,238 did not (the non-SGA group).

We compared the risks of short-term outcomes (neonatal mortality and morbidities), long-term outcomes (growth status, including weight, height, and head circumference <10th percentile, and neurodevelopmental impairments at 2 years of age). Subgroup analysis was performed by stratification of gestation age (GA): GA 24–26, 27–29 and 30–32 weeks.

**Results:**

In the analysis of short-term outcomes, the SGA group had an increased risk of neonatal mortality [adjusted odds ratio (OR) = 2.66, 2.99, and 2.19, respectively] in all GA subgroups in comparison with the non-SGA group (*p* < 0.05). The SGA group had a significantly increased risk of bronchopulmonary dysplasia in GA 27–29 and 30–32 weeks (adjusted OR = 2.11 and 1.86, respectively). We also found that there was an increased risk of severe retinopathy of prematurity in GA 24–26 and 27–29 weeks in the SGA group compared with the non-SGA group (adjusted OR = 1.68 and 1.59, respectively).

In the analysis of long-term outcomes, the SGA group had a significantly increased risk of NDI throughout all GA subgroups (adjusted = 1.94, 1.33, and 1.35, respectively) in comparison with the non-SGA group. The SGA groups also had an increased risk of growth status <10th percentile at 2 years of age (*p* < 0.05).

**Conclusions:**

SGA VLBW premature infants had higher risks of neonatal death, growth status <10th percentile, and NDI at 2 years of corrected age compared with the non- SGA premature infants. Prenatal surveillance, postnatal attention, and long- term follow-up are warranted to improve the outcomes of VLBW SGA premature infants.

## 1. Introduction

Advances in fetomaternal and neonatal medicine have reduced mortality and morbidity rates among very-low-birth-weight (VLBW, i.e., birth weight ≤1,500 g) premature infants in recent decades. The prevalence of preterm birth and VLBW preterm birth were 9.31% and 0.88% in Taiwan between 2012 and 2017, respectively (1). Neurodevelopmental outcomes, which affects the quality of life of both the preterm children and their caregivers, is an increasing focus of pediatric research worldwide (2–4). In neonatal intensive care units, premature infants born with small-for-gestational-age (SGA) status accounted for a significant proportion of all admitted cases (5).

Previously, the two terms “intrauterine growth restriction (IUGR)” and “SGA” were used interchangeably (6–8). However, IUGR and SGA are two different but closely related conditions—-IUGR refers to fetal growth restriction (<10th percentile) before delivery, while SGA is defined as growth retardation at birth, i.e., birthweight <10th percentile (9, 10) or less than 2 standard deviations (SDs) below the mean value for gestational age or age-sex-specific chart (11). Accordingly, several studies have investigated the outcomes of SGA and IUGR neonates in the same report (6, 8, 12, 13).

The etiologies of IUGR and SGA are diverse (14, 15). Toxic substances, tobacco-smoking, perinatal infection, genetic disorders, chromosomal anomalies, maternal preeclampsia, chronic hypertension, maternal absent end-diastolic-velocity (AEDV) or reversed end-diastolic-velocity (REDV) condition, antiphospholipid syndrome and maternal nutritional factors potentially lead to disturbances in fetal growth and subsequent SGA (14, 16–18). Gutbrod et al. reported that VLBW premature SGA neonates seem to have dual risks owing to their shorter duration of GA as well as developmental retardation in the uterus (19). Because being born with SGA status has been regarded an essential risk for neonatal death and morbidities in premature neonates (20–23), timing of delivery was a major concern. However, the impact of SGA status on neonatal morbidity varies. Procianoy et al. stated that a stressful intrauterine environment might lead to enhanced maturation in premature SGA infants; therefore, SGA infants do not have an increased risk of respiratory distress syndrome or intraventricular hemorrhage (24). In contrast, the Vermont Oxford network reported stated that preterm SGA infants had higher risks for neonatal death and morbidities, including necrotizing enterocolitis (25). Studies on the impact of SGA in VLBW preterm neonates on growth and neurodevelopmental outcomes have reported inconsistent findings (13, 15, 23, 26). Two studies investigated the association of the timing of delivery with neonatal morbidities and neurological outcomes at 2 years of age, respectively (7, 27). The most updated information about the outcomes related to SGA status may help prenatal counselling, shared decision-making with parents as well as prediction of long-term outcomes (28).

Heterogeneity exists in growth and neurodevelopmental outcomes in SGA preterm infants compared to those in the non-SGA group. This is due to different definitions of SGA as well as inclusion criteria of the studies. Children born SGA were unable to have catch-up-growth ranging from 13 to 40% in different reports (20). Hokken-Koelega et al. stated that SGA preterm infants may achieve catch-up-growth by 1 to 2 years of age (20). In contrast, Itabashi et al. stated that children with previous SGA status did not show catch-up-growth by 2 years of corrected age (29).

Because of countless prenatal, perinatal and postnatal variables, VLBW premature neonates born SGA are especially at risk of neurodevelopmental impairment (NDI) until later life (15, 23). Many longitudinal follow-up studies have demonstrated that both term and premature SGA neonates are at risk of conditions, such as poorer cognitive skills, impaired motor development, delay in language and learning, unsatisfactory reading ability and more behavioral disorders in childhood and into adolescents compared to their matched appropriate-for-gestational age (AGA) groups (30). However, a few studies have reported no significant difference between SGA and AGA groups regarding cognitive outcomes after adjusting for confounders such as home, educational environment, social background, and educational status of both parents (31).

This study aimed to assess the effects of being born SGA status on short-term outcomes (neonatal mortality and morbidities) and long-term outcomes (growth status <10th percentile and NDI at 2 years of corrected age) among VLBW preterm infants compared with the non-SGA VLBW premature infants.

## 2. Materials and methods

### 2.1. Study cohort and data collection

VLBW premature neonates born between 2012 and 2017 at 22 neonatal care centers were enrolled in the Taiwan Premature Infant Follow-up Network (TPFN) database. This database covers more than 70% of all tertiary medical centers which are capable of caring for VLBW preterm neonates in Taiwan. This prospective, longitudinal, multicenter cohort study evaluated VLBW premature neonates admitted to neonatal wards including intensive care units (NICUs) or newborn general wards within 1 week after birth until they survived to discharge. Sociodemographic, perinatal, and neonatal data during hospitalization were collected. The exclusion criteria were infants with major congenital anomalies or chromosomal disorders. SGA infants were defined as having a birth weight less than the 10th percentile for gestational age (GA) according to Taiwanese infants' sex-specific standards for singleton (9) or twins (10).

This study was approved by the Institutional Review Board of Mackey Memorial Hospital (IRB number 20MMHIS277e). All parents signed an informed consent form designed by the Premature Baby Foundation of Taiwan.

**Figure 1:**
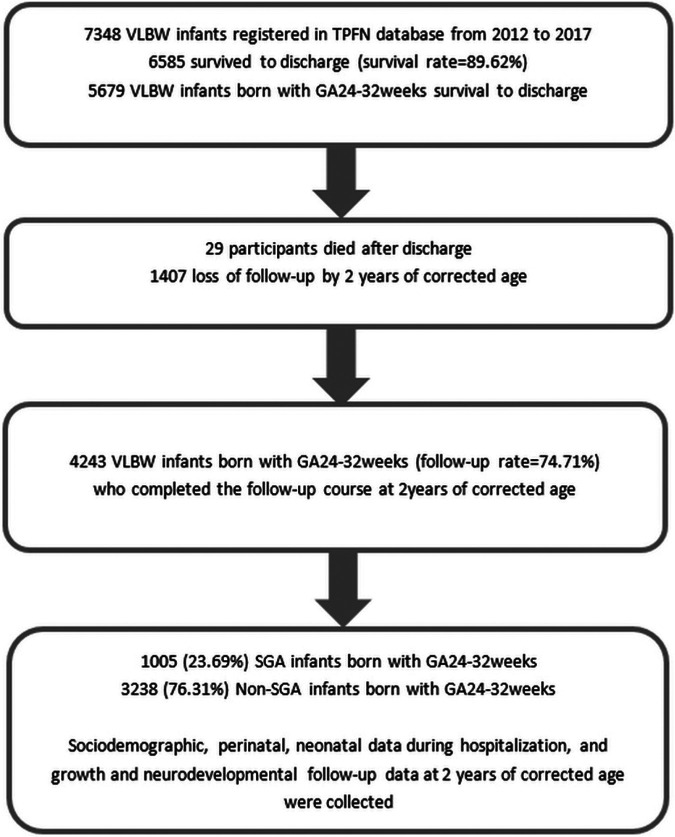
Flow diagram for the study participants.

Infants born with a GA of 24–32 weeks in the TPFN database who survived to discharge and completed follow-up to a corrected age of 2 years were enrolled into the final analysis. Among these participants, those who were SGA were compared with those who were non-SGA in neonatal and 2 years' neurological outcomes. Subgroup analysis was performed by stratifying the SGA and non-SGA groups in to three groups by GA: 24–26, 27–29 and 30–32 weeks. Figure 1 shows the flow diagram of the study participants.

### 2.2. Short-term outcomes: neonatal mortality and morbidities before discharge

Neonatal data, including the details of neonatal morbidities and management during NICU hospitalization, were recorded. Bronchopulmonary dysplasia (BPD) was defined as oxygen requirement at the postmenstrual age of 36 weeks. Severe intraventricular hemorrhage (IVH, ≥grade 3) was diagnosed using brain sonography. Cystic periventricular leukomalacia (cystic PVL) was detected by brain sonography or cranial magnetic resonance imaging (MRI) scan. Necrotizing enterocolitis (NEC) (≥Stage 2A) was defined according to modified Bell's criteria. Advanced retinopathy of prematurity (ROP) (≥Stage III or requiring laser or injection treatment) was classified according to international guidelines.

### 2.3. Long-term outcomes: growth status and NDI at 2 years of corrected age

All study participants were followed until a corrected age of 2 years in the TPFN. Growth status was assessed, including body weight (BW), body length (BL), and head circumference (HC); these parameters <10th percentile at 2 years of corrected age were defined as <10.5 kg, <83.9 cm, and <46.5 cm, respectively, in boys, and <9.9 kg, <82.3 cm, and <45.4 cm, respectively, in girls (32). Neurodevelopmental outcomes were assessed by experienced pediatric neurologists or psychologists. The Bayley Scales of Infant Development, 3rd Edition (BSID-III) was used to evaluate cognitive, language, and motor functions. The following data were recorded at 2 years of corrected age from the TPFN database: cognitive composite score (CCS), language composite score (LCS), and motor composite score (MCS). Cerebral palsy (CP) was defined as a non-progressive motor impairment in non-ambulatory children or those requiring assistive equipment for ambulation at 2 years of corrected age. CP also includes moderate to severe dysfunction of extremities, including spastic hemiparesis, spastic diplegia and spastic tetraparesis. NDI was defined as one or more of the followings: CCS < 85, MCS < 85, cerebral palsy, binocular blindness, hearing impairment corrected with hearing aids, or deafness (33).

### 2.4. Statistical analysis

All statistical analyses were conducted using R version 4.0.2 (R Core Team, 2020). Continuous data were presented as mean and SD, and categorical data were presented as numbers and proportions. Unadjusted comparisons between categorical variables were performed using the chi-square test, and continuous data were compared using the independent t-test. Multivariable logistic regression was used to estimate the relative risk of poor cognitive development in VLBW SGA infants compared with non-SGA preterm infants. Potential confounders including sex, use of prenatal steroids, preterm premature rupture of membranes, being the first-child, and paternal and maternal educational status less than 12 years were included in the model (34–39). Adjusted odds ratios (ORs) and 95% confidence intervals (CIs) were calculated for all outcomes. Type I error rate was set at 5% throughout this study.

## 3. Results

A total of 7,438 VLBW premature neonates born between 2012 and 2017 in 22 neonatal care centers were included in the TPFN database. Among them, 6,585 survived to discharge. Those born at a GA of 24–32 weeks (*n* = 5,679) were enrolled, of whom 29 died after discharge and 1,407 were lost to follow-up by a corrected age of 2 years. The remaining 4,243 VLBW infants completed the follow-up course (follow-up rate = 74.71%), of whom 1,005 (23.69%) had SGA status and 3,238 (76.31%) did not (i.e., non-SGA group) (Figure 1).

### 3.1. Characteristics of the participants

Sociodemographic, perinatal, and neonatal data of the SGA and non-SGA groups were shown in Table 1. Significant differences between the two groups were observed in prenatal, neonatal and sociodemographic factors, including maternal age, preterm premature rupture of membrane (PPROM) ≥18 h, maternal preeclampsia, fetal distress, rates of cesarean-section delivery, single birth, no siblings, and Apgar score <6 at 5 min (*p* < 0.001).

**Table 1:** Sociodemographic, perinatal and neonatal data of the SGA vs. non-SGA groups with a gestational age of 24–32 weeks.

Variables	SGA (*n* = 1,005)	Non-SGA (*n* = 3,238)	*p* value
Gestational age (weeks)	30.2 ± 2.0	28.3 ± 2.0	<0.001
Birth weight (grams)	980 ± 260	1,123 ± 247	<0.001
Gender (Males) (%)	51.44% (=517/1,005)	52.73% (=1,675/3,238)	0.87
Maternal age (years)	33.4 ± 4.9	32.9 ± 4.9	<0.001
Paternal age (years)	35.6 ± 5.6	35.3 ± 5.5	0.29
Maternal education ≤12 years (%)	29.41% (=292/993)	29.99% (=951/3,171)	0.73
Paternal education ≤12 years (%)	32.69% (=304/930)	32.35% (=953/2,946)	0.85
PPROM^a^ ≥18 h(%)	18.01% (=181/1,005)	37.48% (=1,212/3,234)	<0.001
Maternal preeclampsia (%)	50.05% (=503/1,005)	11.41% (=369/3,234)	<0.001
Fetal distress (%)	27.46% (=276/1,005)	10.69% (=346/3,238)	<0.001
Antenatal steroid use (%)	69.62% (=699/1,004)	65.55% (=2,122/3,237)	0.02
Cesarean-section delivery (%)	88.56% (=890/1,005)	65.41% (=2,118/3,238)	<0.001
Single birth (%)	78.19% (=785/1,004)	68.66% (=2,221/3,235)	<0.001
First child, no siblings (%)	65.14% (=654/1,004)	58.00% (=1,878/3,238)	<0.001
Apgar score ≤6 at 5 min (%)	13.30% (=133/1,000)	17.64% (=569/3,226)	<0.001
Acidosis of initial blood gas analysis^b^ (%)	26.54% (=259/976)	27.58% (=877/3,180)	0.52

^a^ PPORM: preterm premature rupture of membranes ≥18 h.

^b^ Acidosis of initial blood gas analysis: (pH < 7.25) of the first arterial blood gas after birth.

### 3.2. Short-term outcomes: neonatal mortality and morbidities before discharge

Subgroup analyses by GA (24–26, 27–29 and 30–32 weeks) were performed to investigate the effects of SGA on the incidence of various neonatal outcomes (Table 2). Multivariable analyses were performed to explore the associations between SGA and short-term outcomes after adjusting for sex, use of prenatal steroids, and preterm premature rupture of membranes based on the literature review.

**Table 2:** Multivariable-adjusted odds ratio (95% confidence interval) of morbidities before discharge in the SGA group (with the non-SGA group as the reference) by gestational age subgroups^a^.

Variables^b^	GA 24–26	GA 27–29	GA 30–32
SGA (*N*)	78	278	649
Non-SGA (*N*)	840	1,593	805
BPD	1.17 (0.68–2.01) (*p* = 0.57)	2.11 (1.62–2.74) (*p* < 0.001)	1.86 (1.39–2.48) (*p* < 0.001)
Severe IVH	0.53 (0.21–1.35) (*p* = 0.18)	1.17 (0.68–2.03) (*p* = 0.57)	0.70 (0.26–1.88) (*p* = 0.48)
Cystic PVL	1.34 (0.58–3.05) (*p* = 0.49)	1.28 (0.75–2.18) (*p* = 0.37)	1.28 (0.64–2.58) (*p* = 0.49)
NEC (≥Stage 2A)	1.23 (0.42–3.61) (*p* = 0.70)	1.56 (0.66–3.69) (*p* = 0.31)	1.63 (0.30–8.77) (*p* = 0.57)
Severe ROP (≥Stage 3 or requiring therapy)	1.68 (1.05–2.68) (*p* = 0.03)	1.59 (1.03–2.45) (*p* = 0.03)	0.86 (0.42–1.73) (*p* = 0.66)
Neonatal death^c^	2.66 (1.88–3.77) (*p* < 0.001)	2.99 (2.14–4.18) (*p* < 0.001)	2.19 (1.27–3.77) (*p* < 0.01)

SGA, small for gestational age; GA, gestational age; BPD, bronchopulmonary dysplasia; IVH, intraventricular hemorrhage; Cystic PVL, cystic periventricular leukomalacia; NEC, necrotizing enterocolitis; ROP, retinopathy of prematurity.

^a^ The non-SGA group was the reference.

^b^ All (except for neonatal death) adjusted for sex, use of prenatal steroids, preterm premature rupture of membranes.

^c^ Univariate logistic regression analysis was used for Neonatal death.

The results showed that the SGA group was associated with an increased risk of death in all GA subgroups (adjusted OR = 2.66, 2.99, and 2.19, respectively) compared with the non-SGA group (*p* < 0.01). Although an increased risk of BPD was observed in all SGA subgroups, the risk was significantly higher only in the GA 27–29 and 30–32 subgroups (adjusted OR = 2.11 and 1.86, respectively, *p* < 0.001). There were also increased risks in cystic PVL and NEC in all SGA subgroups, however none of them were statistically significant. An increased risk of severe ROP requiring treatment was observed in the SGA subgroups with GA at 24–26 and 27–29 weeks (adjusted OR = 1.68 and 1.59, respectively, *p* < 0.05); however, a non-adverse effect was observed in GA 30–32 weeks (adjusted odds ratio = 0.86, *p* = 0.66).

### 3.3. Long-term outcomes: growth status and NDI at 2 years of corrected age

The incidence rates of binocular blindness were 0% (=0/78), 0% (=0/278), and 0.15% (=1/649) in the SGA subgroups (GA24–26, 27–29 and 30–32 weeks), compared to 0.12% (=1/840), 0.06% (=1/1,593), and 0% (=0/805) in the non-SGA subgroups.

The incidences of hearing impairment requiring hearing aids or deafness were 5.13% (=4/78), 2.52% (=7/278), and 0.77% (=5/649) in the SGA subgroups (GA24–26, 27–29 and 30–32 weeks), compared to 2.74% (=23/840), 1.95% (=31/1,593), and 0.99% (=8/805) in the non-SGA subgroups.

Multivariable analyses were performed to explore the association between SGA and neurodevelopmental outcomes at 2 years of corrected age after adjusting for sex, use of prenatal steroids, preterm premature rupture of membranes, being the first-child, maternal education ≤12 years and paternal education ≤12 years according to the literature review. Table 3 showed crude and adjusted ORs with a 95% confidence interval (CI) for growth status and neurodevelopmental outcomes at 2 years of corrected age. The SGA group had a growth status lower than the 10th percentile (i.e., BW, BL, and HC <10th percentile) (*p* < 0.001) (32). SGA status also had a significant impact on NDI compared with non-SGA status (*p* < 0.05).

**Table 3:** Unadjusted and multivariable-adjusted odds ratio (95% confidence interval) of growth status and neurodevelopmental outcomes at 2 years of corrected age in the SGA group (with the non-SGA group as the reference) with a gestational age of 24–32 weeks.

Variables^e^,^f^	Unadjusted analysis				Multivariable adjusted analysis: odds ratios	
	SGA (*n* = 1,005)	Non-SGA (*n* = 3,238)	Crude OR	*p* value	aOR	*p* value
BW < 10th percentile	364/963 (=37.80%)	659/3,128 (=21.07%)	2.28	<0.001	2.25 (1.92–2.65)	<0.001
BL < 10th percentile	391/952 (=41.07%)	808/3,102 (=26.05%)	1.98	<0.001	1.94 (1.66–2.26)	<0.001
HC < 10th percentile	308/901 (=34.18%)	594/2,979 (=19.94%)	2.09	<0.001	2.07 (1.75–2.45)	<0.001
CCS^a^ < 85	160/1,003 (15.95%)	490/3,228 (=15.18%)	1.06	0.55	1.07 (0.87–1.33)	0.49
MCS^b^ < 85	194/1,002 (=19.36%)	574/3,228 (=17.78%)	1.11	0.26	1.13 (0.93–1.37)	0.22
CP^c^	66/1,002 (=6.59%)	215/3,231 (=6.65%)	0.99	0.94	0.96 (0.72–1.32)	0.87
NDI^d^	279/1,005 (=27.76%)	800/3,238 (=24.71%)	1.17	0.05	1.21 (1.02–1.44)	0.03

^a^ CCS: Cognitive composite score.

^b^ MCS: Motor composite score.

^c^ CP: Cerebral palsy.

^d^ NDI: Neurodevelopmental impairment.

^e^ Birth weight (BW) < 10th percentile, Body length (BL) < 10th percentile, Head circumference (HC) < 10th percentile: Adjusted for sex, preterm premature rupture of membranes, use of prenatal steroids.

^f^ Long-term outcomes were adjusted for sex, preterm premature rupture of membranes, use of prenatal steroids, being the first-child, paternal education ≤ 12 years, and maternal education ≤ 12 years, using logistic regression model.

Tables 4–6 showed the results of multivariable analyses of growth status and neurodevelopmental outcomes in the three GA subgroups (24–26, 27–29, and 30–32 weeks). The SGA infants had a growth status less than the 10th percentile (i.e., BW, BL, and HC <10th percentile) in all GA subgroups at 2 years of corrected age (*p* < 0.01).

**Table 4:** Unadjusted and multivariable-adjusted odds ratio (95% confidence interval) of growth status and neurodevelopmental outcomes at 2 years of corrected age in the SGA group (with the non-SGA group as the reference) with a gestational age of 24–26 weeks.

Variables^e^,^f^	Unadjusted analysis				Multivariable adjusted analysis: odds ratios	
	SGA (*n* = 78)	Non-SGA (*n* = 840)	Crude OR	*p* value	aOR	*p* value
BW < 10th percentile	42/73 (=57.53%)	233/812 (=28.69%)	3.37	<0.001	3.33 (2.04–5.45)	<0.001
BL < 10th percentile	38/72 (=52.78%)	273/804 (=33.96%)	2.17	<0.01	2.21 (1.35–3.60)	<0.01
HC < 10th percentile	38/67 (=56.72%)	248/777 (=31.92%)	2.15	<0.01	2.75 (1.65–4.57)	<0.001
CCS^a^ < 85	21/77 (=27.27%)	193/835 (=23.11%)	1.24	0.41	1.35 (0.77–2.35)	0.28
MCS^b^ < 85	29/77 (=37.66%)	219/838 (=26.13%)	1.71	0.03	1.77 (1.06- 2.94)	0.02
CP^c^	9/77 (=11.69%)	75/834 (=8.99%)	1.34	0.44	1.45 (0.68–3.06)	0.33
NDI^d^	36/78 (=46.15%)	291/840 (=34.64%)	1.62	0.04	1.68 (1.03- 2.76)	0.03

^a^ CCS: Cognitive composite score.

^b^ MCS: Motor composite score.

^c^ CP: cerebral palsy.

^d^ NDI: neurodevelopmental impairment.

^e^ Birth weight (BW) < 10th percentile, Body length (BL) < 10th percentile, Head circumference (HC) < 10th percentile: Adjusted for sex, preterm premature rupture of membranes, use of prenatal steroids.

^f^ Long-term outcomes were adjusted for sex, preterm premature rupture of membranes, use of prenatal steroids, being the first-child, paternal education ≤ 12 years, and maternal education ≤ 12 years, using logistic regression model.

**Table 5:** Unadjusted and multivariable-adjusted odds ratio (95% confidence interval) of growth status and neurodevelopmental outcomes at 2 years of corrected age for the SGA group (with the non-SGA group as the reference) with a gestational age of 27–29 weeks.

Variables^e^,^f^	Unadjusted analysis				Adjusted analysis: odds ratios for SGA on the long-term outcomes	
	SGA (*n* = 278)	Non-SGA (*n* = 1,593)	Crude OR	*p* value	aOR	*p* value
BW < 10th percentile	117/267 (=43.82%)	289/1,536 (=18.82%)	3.37	<0.001	3.45 (2.60–4.58)	<0.001
BL < 10th percentile	128/265 (=48.30%)	352/1,522 (=23.13%)	3.11	<0.001	3.19 (2.42–4.21)	<0.001
HC < 10th percentile	95/253 (=37.55%)	242/1,462 (=16.55%)	3.03	<0.001	3.05 (2.26–4.11)	<0.001
CCS^a^ < 85	45/278 (=16.19%)	210/1,590 (=13.21%)	1.27	0.18	1.21 (0.82–1.76)	0.33
MCS^b^ < 85	58/278 (=20.86%)	256/1,588 (=16.12%)	1.37	0.05	1.36 (0.97–1.91)	0.07
CP^c^	16/276 (=5.80%)	105/1,593 (=6.59%)	0.82	0.62	0.84 (0.47–1.51)	0.56
NDI^d^	81/278 (=29.14%)	363/1,593 (=22.79%)	1.39	0.02	1.41 (1.04–1.91)	0.02

^a^ CCS: Cognitive composite score.

^b^ MCS: Motor composite score.

^c^ CP: cerebral palsy.

^d^ NDI: neurodevelopmental impairment.

^e^ Birth weight (BW) < 10th percentile, Body length (BL) < 10th percentile, Head circumference (HC) < 10th percentile: Adjusted for sex, preterm premature rupture of membrane, use of prenatal steroids.

^f^ Long-term outcomes were adjusted for sex, preterm premature rupture of membranes, use of prenatal steroids, being the first-child, paternal education<=12 years, and maternal education<=12 years, using logistic regression model.

**Table 6:** Unadjusted and multivariable-adjusted odds ratio (95% confidence interval) of growth status and neurodevelopmental outcomes at 2 years of corrected age for the SGA group (with the non-SGA group as the reference) with a gestational age of 30–32 weeks .

Variables^e^,^f^	Unadjusted analysis				Adjusted analysis: odds ratios for SGA on the long-term outcomes	
	SGA (*n* = 649)	Non-SGA (*n* = 805)	Crude OR	*p* value	aOR	*p* value
BW < 10th percentile	175/581 (=30.12%)	104/740 (=14.05%)	2.30	<0.001	2.11 (1.63–2.73)	<0.001
BL < 10th percentile	225/615 (=36.59%)	183/776 (=23.58%)	1.86	<0.001	1.73 (1.35–2.20)	<0.001
HC < 10th percentile	175/581 (=30.12%)	104/740 (=14.05%)	2.63	<0.001	2.51 (1.90–3.33)	<0.001
CCS^a^ < 85	94/648 (=14.51%)	87/803 (=10.83%)	1.39	0.04	1.47 (1.04–2.08)	0.03
MCS^b^ < 85	107/647 (=16.54%)	99/802 (=12.34%)	1.41	0.02	1.49 (1.07–2.07)	0.02
CP^c^	41/649 (=6.32%)	35/804 (=4.35%)	1.48	0.10	1.56 (0.94–2.57)	0.08
NDI^d^	162/649 (=24.96%)	146/805 (=18.14%)	1.50	<0.01	1.66 (1.25–2.20)	<0.001

^a^ CCS: Cognitive composite score.

^b^ MCS: Motor composite score.

^c^ CP: cerebral palsy.

^d^ NDI: neurodevelopmental impairment.

^e^ Birth weight (BW) < 10th percentile, Body length (BL) < 10th percentile, Head circumference (HC) < 10th percentile: Adjusted for sex, premature rupture of membranes, use of prenatal steroids.

^f^ Long-term outcomes were adjusted for sex, preterm premature rupture of membranes, use of prenatal steroids, being the first-child, paternal education ≤ 12 years, and maternal education ≤ 12 years, using logistic regression model.

In the analysis of neurodevelopmental outcomes at 2 years of corrected age, the SGA group was associated with an increased risk of NDI in all GA subgroups (adjusted OR = 1.68, 1.41 and 1.66, respectively, *p* < 0.05). Of note, that among SGA VLBW preterm infants, those at GA 24–26 weeks tended to have motor composite score (MCS) <85 (adjusted OR = 1.77, *p* = 0.02) and NDI (adjusted OR = 1.68, *p* = 0.03). In addition, those at GA 30–32 weeks tended to have delays in development in multiple domains: cognitive composite score (CCS) <85 (adjusted OR = 1.47, *p* = 0.03), MCS <85 (adjusted OR = 1.49, *p* = 0.02) and NDI (adjusted OR = 1.66, *p* < 0.001).

## 4. Discussions and conclusions

Our results showed that VLBW SGA preterm infants had significantly increased risks of neonatal death, growth status <10th percentile, and NDI at 2 years corrected age compared to VLBW non-SGA preterm infants.

Considerable controversy exists regarding short- and long-term outcomes when SGA preterm and non-SGA neonates are compared (16, 22, 26). These heterogeneities arise from different study sample size, definition of SGA, inclusion criteria, periods of follow-up, evaluation methods and tools applied to evaluate neurodevelopmental outcomes, and differences in the underlying pathophysiology of SGA birth (14, 16–18, 40).

Similar to previous reports, we found that cesarean delivery rates were slightly higher in the SGA-group, possibly because of the higher rate of fetal distress, leading to iatrogenic preterm delivery (41). Consistent with the study by Gortner et al., we found that maternal preeclampsia was more common in the SGA group than that in the non-SGA group (16). In Table 1, the non-SGA cohort had a significantly higher rate of Apgar score <6 at 5 min of life. A previous study stated that the SGA group might have adapted instead of the pathology in the uterus (42); therefore, the SGA group may not necessarily have a significantly lower Apgar score after birth than the non-SGA group.

### 4.1. Short-term outcomes: neonatal mortality and morbidities before discharge

Consistent with previous reports (16, 22, 43, 44), we found that being born SGA status was associated with an increased risk of mortality (adjusted OR = 2.66, 2.99, and 2.19 for GA at 24–26, 27–29, and 30–32 weeks, respectively) in all GA subgroups (*p* < 0.05). Although the SGA group had an increased risk of BPD in all GA subgroups, the risk was significantly higher only in the GA 27–29 and 30–32 subgroups (Table 2). Although the mechanism for this finding remains unclear, the presence of oxygen metabolites, free radicals, and diminished antioxidant ability of SGA infants have been speculated to play important roles in the development of BPD (16, 40, 43).

Bronchopulmonary dysplasia (BPD) is a neonatal lung problem with multifactorial etiologies, including possible pulmonary ontogenic changes at higher gestational ages related to nutrition or growth in animal model (45, 46), duration of oxygenation use, degree of ventilator support, and malnutrition status. According to previous reports (43, 47–49), premature SGA infants have a higher risk of developing BPD. In our study, the SGA group had significantly higher BPD rates at GA 27–29 and 30–32 weeks (*p* < 0.001), but not at GA 24–26 weeks (*p* = 0.57). Preterm neonates born at GA 24–26 weeks were relatively small and fragile and tended to have a higher risk of developing BPD. Our observation was that the BPD rates were both high in the SGA and non-SGA groups at GA 24–26 weeks (75.64% vs. 72.63%), so no statistical significance existed between the two groups (*p* = 0.54). Additionally, the nutritional status and degree of ventilator support could not be obtained in the TPFN database, and further large-scale surveys are warranted to evaluate the possible mechanisms.

Similar to previous studies (21, 50), we found that the SGA group had increased risks of cystic PVL and NEC compared with the non-SGA group, although without statistical significance (Table 2). Cystic PVL may result from many pathophysiological factors in IUGR status, including fluctuations in blood pressure (51), autoregulation dysfunction of cerebral blood supply, and interference of programming in developing neurons (44, 50). SGA preterm infants seem to suffer from fetal-placental circulation insufficiency to a greater extent than the non-SGA infants in animal models (8), which might explain the relatively higher risk of NEC.

We also found an increased risk of severe ROP requiring treatment in the SGA subgroups (GA 24–26 and 27–29 weeks) compared with the non-SGA subgroup (adjusted OR = 1.68 and 1.59, *p* < 0.05), however there were non-adverse effects in GA 30–32 weeks (adjusted odds ratio = 0.86, *p* = 0.66) (Table 2). This finding is consistent with previous studies (5, 21). The mechanisms underlying ROP are complicated and multifactorial. One possible mechanism is that SGA VLBW preterm infants born at GA <30 weeks tend to have a higher risk in respiratory distress and need oxygen and mechanical ventilator support (21). Further studies are warranted to explore the association between being born with SGA status and ROP.

### 4.2. Long-term outcomes: growth status and NDI at 2 years of corrected age

Consistent with Itabashi et al.’s report (29), we demonstrated that the SGA group failed to achieve somatic catch-up growth in BW, BL, and HC at 2 years of corrected age in all GA subgroups. These results were consistent with those of Gutbrod et al., who reported that the premature SGA infants had lower weight and height by 3 years of age (19). Approximately 2.5%–3.0% of newborn infants were defined as SGA, and 8%–10% of them did not exhibit catch-up growth after birth (52). However, our findings differed from those of Zadik et al. who reported that most children born with IUGR may achieve catch-up growth before 2 years of age (11). The inconsistency between this study and other reports (9, 10, 20, 53) is probably due to the diverse definitions of SGA adopted in different study groups, i.e., <3rd percentile, <5th percentile, or 2 SDs (or more) below the mean for GA. Heterogeneity may also result from different standards of growth charts among different ethnicities or countries (9, 10, 13, 20, 53). Although growth status <10th percentile does not indicate “failure to thrive (FTT)”, it implies that FTT could happen and reminds parents and pediatricians to follow up the growth trajectory of SGA VLBW preterm infants very closely (8, 11, 20, 54).

Our results showed a significantly increased risk of NDI in the SGA subgroup after adjusting for confounding factors in all GA subgroups (GA 24–26, 27–29 and 30–32 weeks) (Tables 4–6). This finding is consistent with those of previous studies (13, 23).

Children born very preterm, especially before a GA of 32 weeks, are at particularly high risk of long-term disabilities because of various prenatal, perinatal, and neonatal factors (15). Male infants have been reported to be more vulnerable to severe fetal growth retardation, adverse neurodevelopmental outcomes and cerebral palsy in some studies (55). Hintz et al. reported the neonatal variables including the postnatal use of steroids, development of BPD, and early- or late- onset sepsis may be associated with poor neurological outcome in male infants (55). Gortner et al. reported that long-term NDI was determined by cystic PVL or severe intraventricular hemorrhage in high-risk neonates (16).

To identify potential associations between neonatal morbidities and neurodevelopmental outcomes at 2 years of corrected age between the SGA and non-SGA groups, we performed a subgroup analysis according to GA at 24–26, 27–29 and 30–32 weeks. SGA VLBW preterm groups had a significantly higher rate of severe ROP ≧ stage3 or requiring treatment in both GA 24–26 and 27–29 weeks. SGA infants also had a higher incidence of BPD at GA 27–29 and 30–32 weeks (*p* < 0.05). Higher rates of cystic PVL and NEC ≧ stage IIA were observed in all SGA subgroups, although the differences were not significant. We also found that all SGA subgroups had a significantly higher incidence of NDI at 2 years of corrected age compared with non-SGA subgroups. Moreover, the SGA group with a GA of 24–26 weeks had a higher rate of MCS <85 in BSID-III than the non-SGA group at 2 years of corrected age (*p* < 0.05). Furthermore, the SGA group with a GA of 30–32 weeks had CCS < 85, MCS <85 in BSID-III, and NDI at 2 years of corrected age (*p* < 0.05).

Three possible explanations for these results are as follows:

First, the impact of being born with SGA status could last until school age, whereas other variables may only have a temporary effect, owing to **s**tructural immaturity, antioxidant effects, or growth hormone deficiency (31, 56). Sacchi et al. reported that the diversity of intrauterine environment leads to a delayed presentation of SGA or a modified subgroup of IUGR (12, 13). It has been hypothesized that SGA preterm neonates tend to have delayed programming of internal organs or systems in the uterus (44).
Second, the increased neonatal morbidities resulting from SGA could have adverse effects on cognitive, motor, vision, hearing or overall NDI toward at least 2 years of age (56). Regev et al. regarded VLBW premature infants born SGA to have a several-fold higher risk of postnatal morbidities. Pathological processes affecting the development of the heart, systemic blood vessels, neurological system and endocrine axis may contribute to some unfavorable outcomes among SGA premature neonates (44). Since the neonatal aspect can be influenced by maternal pathologies such as maternal hypertension, which gives rise to preterm birth and may potentially affect the long-term neurodevelopmental outcomes (57), our report showed that the SGA group had a higher rate of maternal preeclampsia than the non-SGA group (Table 1, *p* < 0.05), which may be associated with the higher risk of NDI in the SGA group. Further large-scale study in maternal pathology and neurodevelopmental outcomes in SGA neonates are warranted.
We found that VLBW SGA preterm infants had significantly higher risks of BPD in GA 27–29 and 30–32 weeks in comparison with the non-SGA groups (Table 2). Malavolti et al. found that severe BPD was an independent predictor of NDI at 2 years of age (56). We also observed that the SGA premature infants had increased risks of ROP in GA 24–26 and 27–29 weeks compared with the non-SGA group (*p* < 0.05). Severe ROP is strongly associated with blindness (58), and Kwinta reported that ROP was a risk factor for psychomotor impairment in VLBW preterm infants (59).
Third, the course of postnatal growth faltering and poor growth status (somatic insufficiency) in SGA preterm infants, especially the persistence of microcephaly reflecting cerebral volume at 2 years of corrected age, has been reported to determine poor intelligence and neurosensory or motor developmental outcomes (2, 23, 60, 61). In agreement with Kato's report (62), we observed that nearly two-fold risk of motor delay in the SGA subgroup with a GA of 24–26 weeks (adjusted OR = 1.77).

It has been proposed that brain maturation was related to retarded motor development in preterm neonates at GA < 28 weeks with severe SGA (62). We found that the SGA infants born at a GA of 30–32 weeks tended to have delays in multiple domains including cognitive and motor composite scores, as well as NDI. This highlights SGA status plays an important role in neurodevelopmental delay in moderately preterm infants. Kato et al. also reported that SGA preterm neonates born before GA of 28 weeks had a higher risk of motor delay (62).

Our results differ from those of other reports (15, 26, 63), which demonstrated that there was no difference between SGA and AGA preterm infants with regard to developmental impairment. A reason for the heterogeneity among different studies may be due to methodological differences and small sample size in different study groups.

Our findings also have important public health implications. The higher risk of NDI in VLBW preterm neonates born SGA indicates that they may need more resources for rehabilitation including occupational, physical, and speech therapies. Additional attention may also be required when this vulnerable group enters school. This study suggests that government health agencies, including the Ministry of Health and Welfare, and the Bureau of National Health Insurance in Taiwan, should provide more resources to SGA VLBW preterm infants to improve their survival rate and neurodevelopmental outcomes. We also suggest that our government health agencies provide more supports for high-risk pregnant women with fetal growth restriction in their prenatal examinations, including Doppler sonographic examinations for umbilical blood flow to detect ADEV or REDV, biophysics, and fetal growth velocity, which provide very important information for the timing of delivery, either iatrogenic delivery or expectant management (41, 64).

The strength of this study is the prospective, multicenter cohort design with the inclusion of 22 neonatal care centers, which covers approximately 70%–80% VLBW preterm neonates born in Taiwan. This is the first population-based cohort study on growth and neurodevelopmental outcomes at 2 years of corrected age in SGA VLBW preterm infants compared with non-SGA VLBW preterm infants in Taiwan. However, this study had some limitations. Although the TPFN database includes very detailed and complete perinatal and neonatal data, the incidence of SGA preterm birth in Taiwan could not be obtained by exploration in this study, and some obstetric sonographic parameters, such as symmetric or asymmetric IUGR status, placental blood flow and fetal cerebral blood flow patterns, were not available. Detailed obstetric sonogram data can help assess fetal growth restriction, which may predispose individuals to SGA.

Another limitation of this study was the case distribution of the cohort. By definition, SGA is the 10th percentile by birthweight, which means it should make up about 10% of the general VLBW population. However, the TPFN databank was not a nationwide population, so the overall case number was not large enough to achieve normal distribution in each GA subgroup. Further large-scale population-based cohort study is warranted.

## 5. Conclusions

This study provides important information for prenatal counseling as well as evidence of the public health impacts of SGA births. Our results showed a strong association between SGA birth status and neonatal mortality in VLBW premature infants born at GA of 24–32 weeks. Being born with SGA status was also associated with increased risks of BW, BL, and HC remaining less than the 10th percentile as well as NDI at 2 years of corrected age. Antenatal close surveillance, postnatal comprehensive care, and long-term follow-up are warranted to improve the outcomes of premature VLBW SGA infants.

## Data Availability

The datasets presented in this article are not readily available because All the data were obtained from the database of Taiwan Premature Infant Follow-up Network (TPFN). Data are not available without the permission of TPFN. Please contact TPFN (pbf@pbf.org.tw) directly if there is a need for the access of the data. Requests to access the datasets should be directed to Taiwan Premature Infant Follow-up Network (TPFN), pbf@pbf.org.tw.

## Coordinators in Taiwan Premature Infant Follow-up Network

Jui-Hsing Chang (National coordinator, Mackay Children's Hospital), Kuo-Inn Tsou (Former national coordinator, Cardinal Tien Hospital); Po-Nien Tsao (Regional coordinator, National Taiwan University Hospital); Shu-Chi Mu (Regional coordinator, Shin Kong Wu Ho-Su Memorial Hospital); Chyong-Hsin Hsu (Regional coordinator, Mackay Children's Hospital); Reyin Lien (Regional coordinator, Chang Gung Memorial Hospital); Hung-Chih Lin (Regional coordinator, China Medical University Hospital); Chien-Chou Hsiao (Regional coordinator, Changhua Christian Hospital); Chao-Ching Huang (Regional coordinator, National Cheng Kung University Hospital); Chih-Cheng Chen (Regional coordinator, Chang Gung Memorial Hospital Kaohsiung Branch).
